# Angiogenin, FGF-α, and IL-36β have higher expression levels in aqueous humor of nAMD patients in comparison to cataract patients

**DOI:** 10.1186/s12886-020-01684-7

**Published:** 2020-10-28

**Authors:** Kailin Chen, Wen Xu, Jiao Zheng, Yupeng Shen, Jian Ma, Zhiqing Chen

**Affiliations:** 1 Eye Center, The Second Affiliated Hospital, Zhejiang University School of Medicine, 88 Jiefang Road, Hangzhou, Zhejiang 310009 P. R. China; 2 Department of Head and Neck Surgery, The Affiliated Sir Run Run Shaw Hospital, Zhejiang University School of Medicine, Hangzhou, Zhejiang P. R. China; 3 Department of Ophthalmology, Ningbo Women and Children’s Hospital, Ningbo, Zhejiang P. R. China

**Keywords:** Neovascular age-related macular degeneration, Aqueous humor, Cytokines, Inflammation, Anti-vascular endothelial growth factor, Ranibizumab

## Abstract

**Background:**

Numerous cytokines have been proven to participate in the pathogenesis of neovascular age-related macular degeneration (nAMD). The present study aimed to investigate the aqueous humor cytokine expression profile in nAMD patients before and after ranibizumab treatments in comparison to cataract patients.

**Methods:**

This prospective study included 20 treatment-naïve nAMD eyes of 20 patients who received three consecutive monthly injections of ranibizumab. Aqueous humor samples were collected before the first (baseline), second (1 month later), and third (2 months later) injections. Controls were 20 age- and gender-matched cataract patients without any other ocular disease. The aqueous concentrations of 28 cytokines were measured using a multiplex bead assay. Central macular thickness (CMT) and maximum retinal thickness (MRT)-3 mm were measured by spectral domain optical coherence tomography (SD-OCT). The greatest linear diameter (GLD) was measured by fundus fluorescein angiography (FA).

**Results:**

Three cytokines in aqueous humor, including angiogenin, interleukin-36β (IL-36β), and fibroblast growth factor-acidic (FGF-α) were significantly higher in nAMD patients in comparison to cataract patients, both before and after two consecutive monthly ranibizumab injections. Compared with the nAMD patients’ basal levels, two consecutive monthly ranibizumab injections effectively reduced the aqueous concentrations of VEGF-A and placental growth factor (PlGF), as well as the values of CMT, MRT-3 mm, and GLD.

**Conclusions:**

Angiogenin, IL-36β, and FGF-α have higher expression levels in nAMD patients in comparison to cataract patients, both before and after 2 months of ranibizumab therapy. These cytokines may have correlations with the pathogenesis of nAMD.

## Background

Age-related macular degeneration (AMD) is a leading cause of blindness in the elderly population. Subfoveal geographic atrophy and choroidal neovascularization (CNV) cause severe visual loss in AMD patients [1]. CNV is the main feature of neovascular AMD (nAMD), in which the formation of new aberrant blood vessels leads to macular edema, hemorrhage, fibrosis, and visual impairment [2]. Although nAMD accounts for only approximately 20% of the overall incidence of AMD, this subtype of patients is most likely to have severe visual loss among AMD patients [3]. Polypoidal choroidal vasculopathy (PCV) is a subtype of nAMD, which is characterized by branching choroidal networks with polyp-like aneurysmal dilation [4].

Drusen deposition, RPE hypertrophy, pigment extrusion, and photoreceptor degeneration followed by the invasion of choroidal blood vessels through the Bruch membrane into the retina all lead to nAMD [5]. Angiogenesis and the development of local inflammatory responses are two major known contributors to nAMD pathogenesis [2, 6]. Vascular endothelial growth factor (VEGF) and many other cytokines are important mediators of inflammatory responses in nAMD patients [2, 7]. Ranibizumab is a monoclonal antibody fragment which binds all isoforms of VEGF-A to suppress both excessive angiogenesis and inflammation, improving the clinical appearance of nAMD patients [8]. However, recurrent or persistent CNV is still very common, despite repeated application of anti-VEGF agents, and the efficacy of only using anti-VEGF drugs is still debatable [9, 10].

Intraocular inflammatory condition of nAMD could be reflected by aqueous humor and vitreous fluid more directly and accurately than serum. Collecting aqueous humor is easier and safer than vitreous fluid. And it is reported that the aqueous levels of various cytokines (such as VEGF) are significantly correlated with their vitreous fluid levels [11]. Studying aqueous cytokines in nAMD patients is feasible and necessary to understand the pathogenesis of nAMD, and could provide ideas to develop new antibody drugs. Previous research has reported that in the aqueous humor of eyes with nAMD, many cytokines are found at higher concentrations than in the eyes of cataract patients. These cytokines include angiogenin [12], monocyte chemoattractant protein-1 (MCP-1) [13], interleukin-8 (IL-8) [13], macrophage inflammatory protein (MIP)-1α [14], macrophage-derived chemokine (MDC) [14], and IFN-γ–inducible protein-10 (IP-10/CXCL10) [14]. A previous study has reported that cytokines, including inflammatory factors, are involved in the pathogenesis of nAMD, and that these inflammatory factors respond to anti-VEGF agents differently [13].

However, many cytokines that have different expressions in the eyes of nAMD remain unrecognized. For example, there are few studies about the concentration of C-C motif chemokine ligand (CCL) in aqueous humor of nAMD patients. To fill these gaps in knowledge, we conducted the current analysis. The purpose of the present study was to compare 28 cytokine levels in the aqueous humor of eyes with nAMD before and after treatments with ranibizumab, in comparison to cataract controls. The differences in aqueous cytokines and clinical appearances caused by ranibizumab treatments were also studied. The 28 cytokines were chosen because they take part in the processes of inflammation, angiogenesis, or chemotaxis, and most of them have not been deeply studied in nAMD patients, except for VEGF-A, which was selected as a positive indicator control and is known to decrease after treatment of ranibizumab.

## Methods

### Study design and approval

This study was a prospective study. Approval for the collection and research of human aqueous humor was permitted by the Ethics Committee of the Second Affiliated Hospital, Zhejiang University School of Medicine. All patients were treated in accordance with the Declaration of Helsinki, and written informed consents were obtained from all participants prior to their participation in the study.

### Inclusion/exclusion criteria

In the present study, 20 eyes of 20 consecutive patients with nAMD and 20 eyes of 20 consecutive patients with age-related cataract were included. All eyes were treatment-naïve.

All patients with nAMD or cataract were examined in a full ophthalmological examination, including best-corrected visual acuity (BCVA) determined as the logarithm of the minimum angle of resolution (logMAR), slit-lamp biomicroscopy, intraocular pressure (IOP) measurement, dilated fundus examination, color fundus photography, and spectral domain optical coherence tomography (SD-OCT). In addition to the above examinations, participates in the nAMD group were examined by fundus fluorescein angiography (FA) and fundus indocyanine green angiography (ICGA). SD-OCT was used to exclude patients with AMD in the cataract group.

The diagnosis of nAMD and the discrimination of type I and II CNV were confirmed by FA. The identification of polypoidal choroidal vasculopathy (PCV) was performed by FA and ICGA. Every patient underwent SD-OCT at every visit. Measured by SD-OCT, retinal thickness (RT) refers to the vertical distance from the inner limiting membrane to the Bruch’s membrane, and central macular thickness (CMT) was defined as the vertical distance from the inner limiting membrane to the Bruch’s membrane, at the central fovea of macula. Maximum retinal thickness (MRT)-3 mm was measured by selecting a point with horizontal distance from the central fovea of macula within 3 mm to maximize the vertical retinal thickness. The greatest linear diameter (GLD) of lesion was measured by FA. Active CNV was defined as leakage seen on FA, as well as new macular intraretinal, subretinal hemorrhage, or fluid on SD-OCT.

The inclusion criteria were: (1) older than 50 years; (2) treatment-naïve nAMD or cataract; (3) absence of concurrent ocular diseases. The exclusion criteria were: (1) pathologic myopia; (2) a history of treatment for nAMD, including intravitreal drug injection, photodynamic therapy, and steroid therapy; (3) previous intraocular surgery, except for cataract surgery (for nAMD patients, the cataract surgery had to have been performed at least 12 months prior to inclusion); (4) active inflammation, diabetes mellitus, use of immunosuppressive drugs and corticosteroids, localized and systemic malignant tumors were all excluded from this study.

### Aqueous humor sample collection

All of the nAMD patients received three consecutive monthly intravitreal injections of 0.5 mg ranibizumab. Aqueous samples were collected at baseline (before the first injection), month 1 (before the second injection), and month 2 (before the third injection). At each injection, approximately 0.15 mL of aqueous humor was collected by anterior chamber limbal paracentesis with a 30-gauge needle, and then the injections of ranibizumab were performed through the pars plana. For cataract patients, around 0.15 mL aqueous humor was taken before cataract surgery by 30-gauge limbal paracentesis. Immediately after collection, each aqueous humor sample was transferred to sterile plastic tubes and stored at − 80 °C until analysis.

### Measurement of cytokines using multiplex analysis

Twenty-eight cytokines in aqueous humor samples were detected with a multiplex cytokine assay kit (R&D Systems, Minneapolis, MN, USA) using Luminex technology on a Bio-Plex MAGPIX system (Bio-Rad, Waltham, MA, USA) according to manufacturers’ instructions. Standard curves for each cytokine were generated (in duplicate) by using the reference set of cytokine concentrations supplied in each kit. To avoid between-run imprecision, we measured cytokines in the samples from all patients in a single run. The sample concentration was calculated using a multi-parameter standard curve for each cytokine.

### Statistical analysis

SPSS software (version 26.0) and R statistical language (version 3.6.0) were used to perform statistical analysis. *P* < 0.05 was deemed to be statistically significant. The statistic graphs were drawn by GraphPad Prism 5.

Patients’ gender were compared using χ^2^ tests. Analyzing by the Kolmogorov-Smirnov tests, patients’ age and the anatomic parameters (including GLD, CMT, and MRT-3 mm) were normally distributed. Therefore, statistical differences in patients’ age between nAMD group and cataract group were determined by the student’s t-test. And the differences of anatomic parameters before and after the treatments of ranibizumab in nAMD patients were analyzed by the repeated measure in general linear model, which were then corrected by Bonferroni’s method for comparisons among three nAMD groups. Also analyzed by Kolmogorov-Smirnov tests, plenty of cytokines don’t follow normal distribution in aqueous humor, so nonparametric statistics were chosen here, statistical significant differences were determined by Friedman tests, which were followed by Bonferroni’s tests for comparisons among patients’ groups, while the method of Benjamini and Hochberg was then used to reduce the false-positive rate in the multiple comparisons of multiple cytokines.

## Results

### Demographic characteristics of the patients

As shown in Table 1, mean ages of nAMD and cataract cases were 74.5 ± 6.9 years (mean ± SD; range, 60–87 years) and 71.4 ± 9.4 years (mean ± SD; range, 54–85 years), respectively (*P* = 0.239). Twelve of the 20 nAMD cases (60%) and 10 of the 20 control cases (50%) were men (*P* = 0.525). In the nAMD group, PCV was found in 4 eyes (20%), type I CNV was found in 6 eyes (30%), type II CNV was found in 10 eyes (50%), and there was no retinal angiomatous proliferation.

**Table 1:** Baseline characteristics of nAMD patients

Variables	nAMD group	Cataract group	***P***-Value
n	20	20	/
Male, n (%)	12 (60%)	10 (50%)	0.525 ^a^
Age, years; mean ± SD	74.5 ± 6.9	71.4 ± 9.4	0.239 ^b^
**Disease type**			
CNV type I, n (%)	6 (30%)	/	/
CNV type II, n (%)	10 (50%)	/	/
PCV, n (%)	4 (20%)	/	/
RAP, n (%)	0 (0)	/	/

^a^χ^2^ test; ^b^Student’s *t*-test. nAMD, neovascular age-related macular degeneration; PCV, polypoidal choroidal vasculopathy; RAP, retinal angiomatous proliferation

### Cytokine concentrations in the aqueous humor of cataract patients vs. nAMD patients before and after the treatments with Ranibizumab

The mean ± SD values of 28 cytokines in the aqueous humor of nAMD patients and cataract control patients are summarized in Table 2.

**Table 2:** Aqueous cytokine concentrations in nAMD patients at baseline, month 1, and month 2 vs. cataract patients

Cytokines	Cataract (***n*** = 20)	nAMD, baseline (***n*** = 20)		nAMD, at month 1 (***n*** = 20)		nAMD, at month 2 (***n*** = 20)		
	Mean ± SD (pg/ml)	Mean ± SD (pg/ml)	***P***-Value (vs. cataract)	Mean ± SD (pg/ml)	***P***-Value (vs. cataract)	Mean ± SD (pg/ml)	***P***-Value (vs. cataract)	***P***-Value (vs. baseline)
**Angiogenin**^a^	24,317.3 ± 4154.7	30,190.6 ± 3476.4	**0.01584**^b^	29,507.4 ± 4350.8	**0.02178**^b^	29,965.3 ± 2967.4	**0.01380**^b^	1
BMP-9^a^	3.0 ± 0.2	3.2 ± 0.2	0.09101	3.2 ± 0.3	**0.02178**^b^	3.1 ± 0.3	0.09036	1
CCL1/I-309^a^	1.0 ± 0.3	1.4 ± 0.24	0.09101	1.7 ± 0.8	0.06525	1.4 ± 0.4	0.23958	1
CCL13/MCP-4^a^	6.6 ± 0.9	7.9 ± 1.5	0.06545	8.0 ± 1.9	**0.03600**^b^	7.5 ± 1.6	0.26987	1
CCL2/MCP-1^a^	660.3 ± 217.6	1029.6 ± 1214.8	0.25202	1080.2 ± 1392.7	0.06825	869.2 ± 630.3	0.26987	1
CXCL10/IP-10^a^	27.3 ± 13.9	48.2 ± 41.2	0.09101	43.8 ± 22.1	**0.03600**^b^	36.5 ± 22.8	0.51063	1
CXCL2/GRO β^a^	121.7 ± 24.2	128.5 ± 11.2	0.16895	128.6 ± 24.3	0.54896	130.6 ± 15.7	**0.03866**^b^	1
CXCL8/IL-8^a^	61.2 ± 11.0	84.4 ± 45.8	**0.03920**^b^	80.2 ± 30.9	0.06825	76.6 ± 16.3	0.05919	1
Endothelin-1^a^	33.4 ± 2.6	37.1 ± 6.6	0.48126	37.8 ± 6.3	**0.02547**^b^	35.8 ± 6.1	1	1
**FGF acidic**^a^	22.7 ± 3.3	26.8 ± 2.3	**0.03920**^b^	28.6 ± 6.7	**0.03600**^b^	28.2 ± 7.6	**0.00369**^b^	1
IL-15^a^	4.9 ± 1.8	6.1 ± 1.7	0.16895	6.4 ± 2.5	0.06525	6.0 ± 1.9	0.95610	1
IL-22^a^	21.0 ± 3.8	23.4 ± 2.9	0.09101	24.0 ± 3.6	**0.03394**^b^	23.0 ± 2.6	0.11419	1
**IL-36 β/IL-1F8**^a^	1.1 ± 0.7	1.5 ± 0.3	**0.03920**^b^	1.6 ± 0.7	**0.02613**^b^	1.5 ± 0.4	**0.01634**^b^	1
PDGF-BB^a^	1.7 ± 0.5	2.4 ± 1.0	0.06545	2.3 ± 0.9	**0.03600**^b^	2.1 ± 0.6	0.47894	1
**PlGF**^a^	4.9 ± 0.5	5.3 ± 0.9	0.06545	4.9 ± 1.1	1	4.7 ± 1.2	1	**0.02070**^b^
TNF-α^a^	2.1 ± 0.6	2.4 ± 0.4	0.16895	2.8 ± 1.3	**0.02613**^b^	2.4 ± 0.5	0.70739	1
**VEGF-A**^a^	72.0 ± 21.6	70.6 ± 30.5	1	22.5 ± 1.7	**0.00010**^b^	22.3 ± 1.0	**2.88 × 10**^**−5**b^	**0.00010**^b^
VEGF-C^a^	303.3 ± 47.0	327.0 ± 24.0	0.05820	329.5 ± 39.0	**0.03600**^b^	317.5 ± 35.2	0.45039	1
Angiopoietin-1	105.4 ± 35.3	127.1 ± 67.6	–	126.2 ± 76.1	–	116.4 ± 56.2	–	–
Angiopoietin-2	84.2 ± 16.8	107.1 ± 56.5	–	104.2 ± 57.9	–	101.8 ± 60.7	–	–
CCL20/MIP-3 α	20.9 ± 12.1	20.4 ± 10.7	–	17.8 ± 8.9	–	15.5 ± 7.7	–	–
CCL27/CTACK	4.5 ± 2.1	5.5 ± 1.9	–	5.5 ± 2.2	–	5.2 ± 2.4	–	–
CRP	49,709.8 ± 186,176.6	7566.9 ± 9756.9	–	7132.9 ± 8391.2	–	9574.2 ± 20,248.2	–	–
CXCL16	609.5 ± 132.1	740.2 ± 314.7	–	759.4 ± 324.7	–	739.9 ± 322.5	–	–
Endocan/ESM-1	113.8 ± 35.3	127.6 ± 46.1	–	132.4 ± 51.0	–	126.0 ± 58.0	–	–
FGF basic	17.4 ± 5.3	17.6 ± 6.0	–	16.9 ± 6.5	–	14.5 ± 3.9	–	–
IL-6	8.9 ± 24.1	3.3 ± 5.5	–	6.1 ± 17.3	–	3.9 ± 3.8	–	–
PDGF-AA	37.6 ± 8.8	42.2 ± 10.7	–	41.6 ± 13.5	–	41.1 ± 12.9	–	–

The concentrations were presented as mean ± SD. ^a^These eighteen cytokines which have statistically significant differences among patients’ groups were screened by Friedman tests. Friedman tests were followed by Bonferroni’s tests for comparisons among four patients’ groups, while the method of Benjamini and Hochberg was then used to reduce the false-positive rate in the multiple comparisons of the eighteen cytokines. ^b^Statistical significant differences were found

**Fig. 1:**
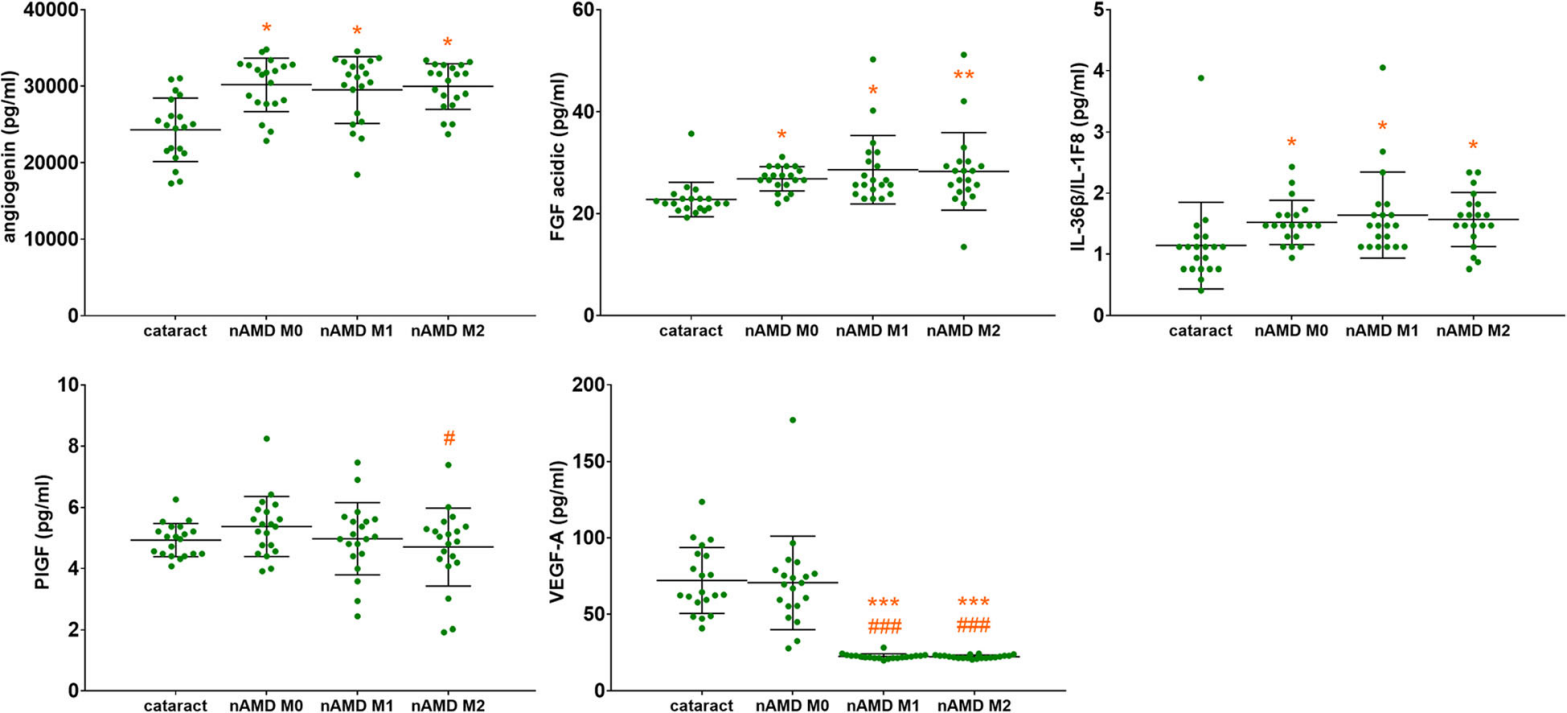
Aqueous humor cytokines concentrations in patients with cataract or nAMD. The nAMD patients received three consecutive monthly ranibizumab injections. Values represent mean ± SD of aqueous levels of cytokines concentrations in 20 cataract or 20 nAMD patients at baseline (M0), month 1 (M1), and month 2 (M2). Aqueous levels of angiogenin, FGF-α, and IL-36β in nAMD patients were significantly higher than in cataract patients, both before and after injections of ranibizumab. Aqueous VEGF-A and PlGF in nAMD patients were significantly reduced after two consecutive monthly ranibizumab injections. *P < 0.05, **P < 0.01, ***P < 0.001 vs. cataract group; ^#^*P* < 0.05, ^##^*P* < 0.01, ^###^*P* < 0.001 vs. nAMD baseline group

Before and after the treatments with ranibizumab, aqueous levels of three cytokines in nAMD patients were significantly higher than those in cataract patients (Table 2 and Fig. 1). These cytokines (*P* = nAMD baseline vs. cataract) are: angiogenin (*P* = 0.01584), fibroblast growth factor-acidic (FGF-α) (*P* = 0.03920), and IL-36β (*P* = 0.03920). The aqueous concentrations of VEGF-A (*P* < 0.001) and placental growth factor (PlGF) (*P* = 0.02070) in nAMD patients were significantly reduced after two consecutive monthly ranibizumab injections, in comparison to baseline levels. However, in the present study, aqueous VEGF-A levels had no significant difference between nAMD patients and cataract patients.

### Anatomic parameters of nAMD patients before and after the treatments with Ranibizumab

SD-OCT and FA data of 16 nAMD patients were available at all three injections (Supplementary Table S1). After two consecutive monthly ranibizumab injections but before the third ranibizumab injection, CMT (*P* < 0.001), MRT-3 mm (*P* < 0.001), and GLD (*P* < 0.001) were all significantly reduced compared to baseline values.

From our data of FA, ICGA, and SD-OCT which were not shown, the activity of CNV in the nAMD group was low. The extents of visual recovery and retinal thickness improvement were not large, also suggesting that most of the nAMD patients were in inactive chronic states.

## Discussion

Pathologic circumstances, such as ischemia, hypoxia, or inflammation, were proangiogenic factors and could lead to the formation of CNV, corresponding cytokines take part in these processes. The present study investigated 28 relevant aqueous cytokines in nAMD patients and cataract patients. Before and after two consecutive monthly ranibizumab injections, the aqueous concentrations of three cytokines, including angiogenin, FGF-α, and IL-36β were significantly higher in eyes with nAMD compared to cataract eyes. As far as we know, it is the first time that the aqueous levels of IL-36β in nAMD patients have been studied. These consequences need to be confirmed and further studied.

Two consecutive monthly ranibizumab injections effectively reduced the aqueous concentrations of VEGF-A and PlGF in nAMD patients, compared with their basal levels. Consistent with the majority of previous researches [8], our study also found that ranibizumab injections reduced CMT, MRT-3 mm, and GLD. The high aqueous levels of the three cytokines in nAMD patients didn’t decrease with the injection of ranibizumab, because they are not the target of ranibizumab, it also reflects that these factors are steadily increased in nAMD eyes, not a coincidence.

In the current study, higher aqueous levels of inflammatory cytokine IL-36β in eyes with nAMD than cataract eyes were detected. Prolonged inflammation is usually detrimental and participates in nAMD [7]. Elevated expression of IL-36β was found in the aqueous humor of acute uveitis, which was involved in the autoreactive T-cell immune response [15]. The aqueous levels of IL-36β in nAMD patients were studied for the first time in the present study.

It is known that angiogenin and FGF-α participate in the process of angiogenesis. A previous study reported that aqueous angiogenin levels were significantly higher in the exudative AMD group than the cataract group [12], but another study found there was no difference in aqueous angiogenin levels between cataract and AMD patients [16]. Angiogenin could activate vessel endothelial cells and smooth muscle cells to facilitate angiogenesis, cell invasion, proliferation, and the formation of tubular structures [17]. Human choroid and retina cells synthesize and internalize angiogenin, which is localized to normal and pathologic vasculature in eyes with AMD [18]. Our study confirms the high expression of angiogenin in the aqueous humor of nAMD patients. FGF-α promotes angiogenesis and inflammation [19, 20]. FGF receptor signaling pathway in endothelial cells plays a critical role in diseases associated with aberrant vascular proliferation including nAMD [21]. The FGF/FGF receptor system could be a target for the development of anti-angiogenic therapies [20]. In a previous study, there was no significant difference in the aqueous FGF-α levels between the nAMD group and control group, but FGF-α concentrations in nAMD patients increased significantly after the intravitreal injection of bevacizumab [22]. In our study, FGF-α concentrations were significantly higher in nAMD patients before and after two consecutive monthly ranibizumab injections, in comparison to cataract patients, which deserves further study.

In the present study, VEGF-A, VEGF-C, and PlGF in the VEGF family were tested. VEGF-A, also called VEGF, exhibits a strong pro-angiogenic effect through binding to VEGFR-1 and VEGFR-2 [23–25]. VEGF-A could be secreted not only by endothelial cells [23, 24, 26] but also by RPE cells [27] and Müller cells in the retina [28], in response to oxygen deprivation, which is one of the most likely reasons leading to the formation of CNV. Anti-VEGF-A medication for neovascular eyes has revolutionized treatment for nAMD patients and preserved their vision [29]. Some previous studies reported higher VEGF-A levels in the aqueous humor of patients with nAMD than in those with cataract [13, 30]. However, some other studies found no significant difference in the aqueous VEGF-A levels between cataract patients and active nAMD patients, or between cataract patients and early nAMD patients [16, 31, 32]. We found aqueous VEGF-A levels had no significant difference between nAMD patients and cataract patients in the present study. There were several reasons, first, in the present study, most of the nAMD cases were in inactive chronic states. Aqueous VEGF-A levels are positively related to the activity of CNV [16, 33]. Consistent with our conclusion, Muether et al. reported that the recurrence of CNV activity shown by SD-OCT is always preceded by an increase of aqueous VEGF and usually followed by loss of visual acuity in the further course [34]. Second, in the present study, half of our patients were type I CNV and PCV. Tong et al. found that VEGF levels in eyes with PCV were significantly lower than those in eyes with CNV [35]. It may reflect that PCV and other nAMD subtypes have different angiogenesis courses. Type I CNV, located below the RPE layer, may lead to the low aqueous VEGF level. However, whether VEGF-A is elevated in the aqueous humor of nAMD patients still needs further investigation.

The main limitation of the current study is the sample size was too small to perform subgroup analysis for differences in VEGF-A or other cytokine levels at baseline, and how these change with time. Second, the observation period was short. Thus, more patients are needed to be followed up for a longer time to confirm the roles of these cytokines in the pathogenesis of nAMD. Collecting aqueous humor of cataract patients as controls instead of healthy volunteers was another limitation of the current study.

## Conclusion

The present study suggests that aqueous angiogenin, FGF-α, and IL-36β levels in nAMD eyes were significantly higher than in cataract eyes, both before and after injections of ranibizumab. These results may further confirm the inflammation-related pathogenesis of nAMD. Therefore, these three cytokines could be novel therapeutic targets for nAMD.

## Supplementary information

**Additional file 1.**

## Data Availability

The datasets analyzed in the current study are available from the corresponding author for reasonable requests.

## Abbreviations

BCVA: Best corrected visual acuity
CCL: C-C motif chemokine ligand
CMT: Central macular thickness
CNV: Choroidal neovascularization
CXCL: C-X-C motif chemokine ligand
FA: (fundus) Fluorescein angiography
FGF-α: Fibroblast growth factor-acidic
GLD: Greatest linear diameter
ICGA: (fundus) Indocyanine green angiography
IL: Interleukin
IOP: Intraocular pressure
IP-10: IFN-γ–inducible protein-10
logMAR: Logarithm of the minimum angle of resolution
MCP-1: Monocyte chemoattractant protein-1
MDC: Macrophage-derived chemokine
MIP: Macrophage inflammatory protein
MRT: Maximum retinal thickness
nAMD: Neovascular age-related macular degeneration
PCV: Polypoidal choroidal vasculopathy
RT: Retinal thickness
SD-OCT: Spectral domain optical coherence tomography
PlGF: placental growth factor
VEGF: Vascular endothelial growth factor

## References

[1] Schmidt-Erfurth U, Chong V, Loewenstein A, Larsen M, Souied E, Schlingemann R (2014). Guidelines for the management of neovascular age-related macular degeneration by the European Society of Retina Specialists (EURETINA). Br J Ophthalmol.

[2] Ambati J, Atkinson JP, Gelfand BD (2013). Immunology of age-related macular degeneration. Nat Rev Immunol.

[3] Balasubramanian SA, Krishna Kumar K, Baird PN (2014). The role of proteases and inflammatory molecules in triggering neovascular age-related macular degeneration: basic science to clinical relevance. Transl Res.

[4] Spaide RF, Yannuzzi LA, Slakter JS, Sorenson J, Orlach DA (1995). Indocyanine green videoangiography of idiopathic polypoidal choroidal vasculopathy. Retina (Philadelphia, Pa).

[5] Ambati J, Ambati BK, Yoo SH, Ianchulev S, Adamis AP (2003). Age-related macular degeneration: etiology, pathogenesis, and therapeutic strategies. Surv Ophthalmol.

[6] Nowak JZ (2006). Age-related macular degeneration (AMD): pathogenesis and therapy. Pharmacol Rep.

[7] Kauppinen A, Paterno JJ, Blasiak J, Salminen A, Kaarniranta K (2016). Inflammation and its role in age-related macular degeneration. Cell Mol Life Sci.

[8] Solomon SD, Lindsley K, Vedula SS, Krzystolik MG, Hawkins BS (2019). Anti-vascular endothelial growth factor for neovascular age-related macular degeneration. Cochrane Database Syst Rev.

[9] Chang AA, Li H, Broadhead GK, Hong T, Schlub TE, Wijeyakumar W (2014). Intravitreal aflibercept for treatment-resistant neovascular age-related macular degeneration. Ophthalmology..

[10] Saito M, Kano M, Itagaki K, Oguchi Y, Sekiryu T (2014). Switching to intravitreal aflibercept injection for polypoidal choroidal vasculopathy refractory to ranibizumab. Retina (Philadelphia, Pa).

[11] Funatsu H, Yamashita H, Noma H, Mimura T, Nakamura S, Sakata K (2005). Aqueous humor levels of cytokines are related to vitreous levels and progression of diabetic retinopathy in diabetic patients. Graefes Arch Clin Exp Ophthalmol.

[12] Agawa T, Usui Y, Wakabayashi Y, Okunuki Y, Juan M, Umazume K (2014). Profile of intraocular immune mediators in patients with age-related macular degeneration and the effect of intravitreal bevacizumab injection. Retina (Philadelphia, Pa).

[13] Motohashi R, Noma H, Yasuda K, Kotake O, Goto H, Shimura M (2017). Dynamics of inflammatory factors in aqueous humor during Ranibizumab or Aflibercept treatment for age-related macular degeneration. Ophthalmic Res.

[14] Agrawal R, Balne PK, Wei X, Bijin VA, Lee B, Ghosh A (2019). Cytokine profiling in patients with exudative age-related macular degeneration and Polypoidal Choroidal vasculopathy. Invest Ophthalmol Vis Sci.

[15] Garlanda C, Dinarello CA, Mantovani A (2013). The interleukin-1 family: back to the future. Immunity..

[16] Muether PS, Neuhann I, Buhl C, Hermann MM, Kirchhof B, Fauser S (2013). Intraocular growth factors and cytokines in patients with dry and neovascular age-related macular degeneration. Retina (Philadelphia, Pa).

[17] Gao X, Xu Z (2008). Mechanisms of action of angiogenin. Acta Biochim Biophys Sin.

[18] Skeie JM, Zeng S, Faidley EA, Mullins RF (2011). Angiogenin in age-related macular degeneration. Mol Vis.

[19] Zakrzewska M, Marcinkowska E, Wiedlocha A (2008). FGF-1: from biology through engineering to potential medical applications. Crit Rev Clin Lab Sci.

[20] Rusnati M, Presta M (2007). Fibroblast growth factors/fibroblast growth factor receptors as targets for the development of anti-angiogenesis strategies. Curr Pharm Des.

[21] Oladipupo SS, Smith C, Santeford A, Park C, Sene A, Wiley LA (2014). Endothelial cell FGF signaling is required for injury response but not for vascular homeostasis. Proc Natl Acad Sci U S A.

[22] Cabral T, Lima LH, Mello LGM, Polido J, Correa EP, Oshima A (2018). Bevacizumab injection in patients with Neovascular age-related macular degeneration increases Angiogenic biomarkers. Ophthalmol Retina.

[23] Rosen LS (2002). Clinical experience with angiogenesis signaling inhibitors: focus on vascular endothelial growth factor (VEGF) blockers. Cancer Control.

[24] Yamazaki Y, Morita T (2006). Molecular and functional diversity of vascular endothelial growth factors. Mol Divers.

[25] Shibuya M (2011). Vascular endothelial growth factor (VEGF) and its receptor (VEGFR) signaling in angiogenesis: a crucial target for anti- and pro-Angiogenic therapies. Genes Cancer.

[26] Takahashi H, Shibuya M (2005). The vascular endothelial growth factor (VEGF)/VEGF receptor system and its role under physiological and pathological conditions. Clin Sci (London, England : 1979).

[27] Klettner A, Westhues D, Lassen J, Bartsch S, Roider J (2013). Regulation of constitutive vascular endothelial growth factor secretion in retinal pigment epithelium/choroid organ cultures: p38, nuclear factor kappaB, and the vascular endothelial growth factor receptor-2/phosphatidylinositol 3 kinase pathway. Mol Vis.

[28] Wang JJ, Zhu M, Le YZ (2015). Functions of Muller cell-derived vascular endothelial growth factor in diabetic retinopathy. World J Diabetes.

[29] Campbell M, Doyle SL (2019). Current perspectives on established and novel therapies for pathological neovascularization in retinal disease. Biochem Pharmacol.

[30] Sato T, Takeuchi M (2018). Intraocular inflammatory cytokines in patients with neovascular age-related macular degeneration before and after initiation of intravitreal injection of anti-VEGF inhibitor.

[31] Ng DS, Yip YW, Bakthavatsalam M, Chen LJ, Ng TK, Lai TY (2017). Elevated angiopoietin 2 in aqueous of patients with neovascular age related macular degeneration correlates with disease severity at presentation. Sci Rep.

[32] Rezar-Dreindl S, Sacu S, Eibenberger K, Pollreisz A, Buhl W, Georgopoulos M (2016). The intraocular cytokine profile and therapeutic response in persistent Neovascular age-related macular degeneration. Invest Ophthalmol Vis Sci.

[33] Roh MI, Kim HS, Song JH, Lim JB, Koh HJ, Kwon OW (2009). Concentration of cytokines in the aqueous humor of patients with naive, recurrent and regressed CNV associated with amd after bevacizumab treatment. Retina (Philadelphia, Pa).

[34] Muether PS, Hermann MM, Viebahn U, Kirchhof B, Fauser S (2012). Vascular endothelial growth factor in patients with exudative age-related macular degeneration treated with ranibizumab. Ophthalmology..

[35] Tong JP, Chan WM, Liu DT, Lai TY, Choy KW, Pang CP (2006). Aqueous humor levels of vascular endothelial growth factor and pigment epithelium-derived factor in polypoidal choroidal vasculopathy and choroidal neovascularization. Am J Ophthalmol.

