# Supplementary material for: Angiogenin, FGF-α, and IL-36β have higher expression levels in aqueous humor of nAMD patients in comparison to cataract patients
Source: BMC Ophthalmol. 2020 Oct 28;20:431. doi: 10.1186/s12886-020-01684-7 (PMC7592502; doi:10.1186/s12886-020-01684-7)
Supplement: Supplementary file 1 — Additional file 1. [file 12886_2020_1684_MOESM1_ESM.docx]

Supplementary Table S1. Anatomic parameters of nAMD patients at baseline, month 1, and month 2.

| **Variables** | **nAMD, baseline (n=16)** | **nAMD, month 1 (n=16)** | | **nAMD, month 2 (n=16)** | |
| --- | --- | --- | --- | --- | --- |
|  | **Mean ± SD or Rate** | **Mean ± SD or Rate** | ***P*-Value (vs. baseline)** | **Mean ± SD or Rate** | ***P*-Value (vs. baseline)** |
| GLD (mm) | 5.49 ± 1.67 | 3.94 ± 1.72 | **<0.005** * | 3.34 ± 1.24 | **<0.001** * |
| CMT (μm) | 481.25 ± 263.09 | 330.63 ± 216.66 | **<0.001** * | 300.5 ±196.6 | **<0.001** * |
| MRT-3mm (μm) | 652.13 ± 259.72 | 507.06 ± 190.93 | **<0.001** * | 477.31 ± 184.15 | **<0.001** * |

GLD, greatest linear diameter; CMT, central macular thickness; MRT-3mm, maximum retinal thickness in the range of 3mm radius centered on the macula. GLD of the lesion was measured by fundus fluorescein angiography (FA). CMT and MRT-3mm were measured by spectral domain optical coherence tomography (SD-OCT). *Analyzed by the repeated measure in general linear model, corrected by Bonferroni’s method for multiple comparisons among three nAMD groups.
